# Supplementary material for: Automated EEG-Based Brainwave Analysis for the Detection of Postoperative Delirium Does Not Result in a Shorter Length of Stay in Geriatric Hip Fracture Patients: A Multicentre Randomized Controlled Trial
Source: J Clin Med. 2024 Nov 20;13(22):6987. doi: 10.3390/jcm13226987 (PMC11595407; doi:10.3390/jcm13226987)
Supplement: Supplementary file 1 [file jcm-13-06987-s001.zip › jcm-3274243-supplementary.pdf]

Journal of
Clinical Medicine

MDPI

Supplementary Table S1: a: Delta Scan scores for patients with and without delirium; b: DOS scores for patients with and without delirium

(a)				
**Measurement**	**Missing n (%)**	**Delirium (n=37; 19%)** *Median; (IQR)*	**No delirium (n=157; 81%)** *Median; (IQR)*	***p*-value**
Day 1 Morning	62 (32)	3 (1.25-4)	1 (1-3)	<0.001
Day 1 Evening	14 (7)	3 (1-5)	1 (1-3)	<0.001
Day 2 Morning	16 (8)	3 (2-4)	2 (1-3)	0.001
Day 2 Evening	15 (8)	3 (2-4)	1 (1-3)	<0.001
Day 3 Morning	19 (10)	3 (1-4)	1 (1-3)	0.004
Day 3 Evening	40 (21)	2 (1-4)	1 (1-3)	0.028
**(b)**				
**Measurement**	**Missing n (%)**	**Delirium (n=33; 17%)** *Median (IQR)*	**No delirium (n=161; 83%)** *Median; (IQR)*	***p*-value**
Day 1 Morning	77 (40)	1 (0-2.5)	0 (0-0)	<0.001
Day 1 Evening	6 (3)	2 (0-5)	0 (0-0)	<0.001
Day 2 Morning	11 (6)	1.5 (0-4.75)	0 (0-0)	<0.001
Day 2 Evening	7 (4)	2 (0-5)	0 (0-0)	<0.001
Day 3 Morning	12 (6)	1 (0-3)	0 (0-0)	<0.001
Day 3 Evening	27 (14)	1 (0-3)	0 (0-0)	<0.001
